# A Safer Prescription: Quality Improvement in Medication Practices on West Ward

**DOI:** 10.1192/bjo.2025.10368

**Published:** 2025-06-20

**Authors:** Mehtab Rahman, Ghazi Seekolu Jhansi, Somya Pandey, Shainy Christopher

**Affiliations:** Cygnet Hospital Harrow, London, United Kingdom

## Abstract

**Aims:** To reduce medication errors on West Ward, a busy adult mental health ward, by addressing multiple domains of medication safety identified in a baseline audit. The project aimed to improve prescribing practices, medication administration, and related processes through targeted interventions and continuous monitoring.

**Methods:** A baseline audit of medication practices on West Ward revealed significant errors across various domains, including temperature recording, medication stock management, MHRA actions and alerts, record keeping, incomplete processes, prescribing technicalities, clinical issues, administration errors, controlled drug management, emergency drug and equipment availability, medicine ordering, and medicine information.

A quality improvement (QI) project was implemented over six months, incorporating three Plan-Do-Study-Act (PDSA) cycles. Interventions included:

Training: Targeted training for doctors and nurses on best practices in medication safety, focusing on identified error hotspots.

Documentation Improvement: Introduction of standardised templates and improved documentation processes to enhance clarity and completeness.

Induction Changes: Revision of the induction process for new staff to emphasise medication safety protocols and ward-specific procedures.

Controlled Drug Review: A comprehensive review and strengthening of controlled drug management procedures, including prescribing, storage, and administration.

MHRA Record Keeping Review: Implementation of a robust system for recording and acting upon MHRA alerts and drug safety information.

Data was collected throughout the project using regular audits of medication practices, mirroring the baseline audit. Error rates were tracked across all targeted domains for each PDSA cycle to assess the impact of the interventions. Sustained improvement was evaluated through follow-up audits after the project’s completion.

**Results:** The QI project demonstrated a significant reduction in medication errors on West Ward. Overall, a 51% reduction in the total number of medication errors was achieved over the six-month period. Each PDSA cycle contributed to this improvement, with error rates progressively decreasing. Specific areas showing marked improvement included prescribing technicalities, administration errors, and controlled drug management. Follow-up audits conducted after the project’s conclusion indicated that the reduced error rates were sustained over time, demonstrating the effectiveness of the interventions.

**Conclusion:** This QI project successfully reduced medication errors on West Ward through a multifaceted approach targeting multiple domains of medication safety. The combination of training, documentation improvements, process changes, and focused reviews. This project demonstrates that targeted QI initiatives can lead to significant and lasting improvements in medication safety within a busy mental health setting, ultimately benefiting patient care and safety. Further work will focus on exploring the factors contributing to sustained improvement and disseminating these findings to other wards and healthcare settings across the organisation.

